# Identification of *pyrC* gene as an immunosuppressive factor in *Francisella novicida* infection

**DOI:** 10.3389/fcimb.2022.1027424

**Published:** 2022-10-26

**Authors:** Takemasa Nakamura, Takashi Shimizu, Ryo Ikegaya, Akihiko Uda, Kenta Watanabe, Masahisa Watarai

**Affiliations:** ^1^ Joint Faculty of Veterinary Medicine, Laboratory of Veterinary Public Health, Yamaguchi University, Yamaguchi, Japan; ^2^ Department of Veterinary Science, National Institute of Infectious Diseases, Tokyo, Japan

**Keywords:** *Francisella*, tularemia, pyrimidine, immune response, cytokine

## Abstract

*Francisella tularensis*, a bacterial causative agent of the zoonosis tularemia, is highly pathogenic to humans. The pathogenicity of this bacterium is characterized by intracellular growth in immune cells, like macrophages, and host immune suppression. However, the detailed mechanism of immune suppression by *F*. *tularensis* is still unclear. To identify the key factors causing *Francisella*-mediated immunosuppression, large-scale screening using a transposon random mutant library containing 3552 mutant strains of *F*. *tularensis* subsp*. novicida* (*F. novicida*) was performed. Thirteen mutants that caused stronger tumor necrosis factor (TNF)-α production in infected U937 human macrophage cells than the wild-type *F*. *novicida* strain were isolated. Sequencing analysis of transposon insertion sites revealed 10 genes, including six novel genes, as immunosuppressive factors of *Francisella*. Among these, the relationship of the *pyrC* gene, which encodes dihydroorotase in the pyrimidine biosynthesis pathway, with *Francisella*-mediated immunosuppression was investigated. The *pyrC* deletion mutant strain (Δ*pyrC*) induced higher TNF-α production in U937 host cells than the wild-type *F*. *novicida* strain. The Δ*pyrC* mutant strain was also found to enhance host interleukin-1β and interferon (IFN)-β production. The heat-inactivated Δ*pyrC* mutant strain could not induce host TNF-α production. Moreover, the production of IFN-β resulting from Δ*pyrC* infection in U937 cells was repressed upon treatment with the stimulator of interferon genes (STING)-specific inhibitor, H-151. These results suggest that *pyrC* is related to the immunosuppressive activity and pathogenicity of *Francisella via* the STING pathway.

## Introduction

Tularemia is a vector-borne zoonosis with severe symptoms, including fever, lymphadenitis, cutaneous lesions, and primary pulmonary involvement in humans (Petersen and Schriefer, 2005). *Francisella tularensis*, the causative agent of tularemia, is a gram-negative, facultative, and intracellular bacterial pathogen. Tularemia is highly contagious, with subcutaneous infection with as few as 10 bacterial cells and aerosol-mediated inhalation of as few as 25 bacterial cells being able to cause human infection (Sjöstedt, 2007). Accordingly, highly virulent *F*. *tularensis* strains have been classified as a Category-A agents of potential bioterrorism by the Centers for Disease Control and Prevention and are required to be handled and contained in BSL-3 laboratories (Shapiro and Schwartz, 2002). *F*. *tularensis* is divided into four subspecies, subsp. *tularensis*, *holarctica*, *mediasiatica*, and *novicida*. Among them, only *Francisella tularensis* subsp. *tularensis* (*F*. *tularensis*) and *Francisellatularensis* subsp. *holarctica* (*F*. *holarctica*) cause tularemia in human (Maurin, 2015). *F*. *novicida* infection in humans is considerably rare and can be handled in a BSL-2 laboratory. Most cases of human *F*. *novicida* infection have involved patients who were immunocompromised or had an underlying disease. However, *F*. *novicida* has over 98% identity to *F*. *tularensis* at the DNA level and shows many characteristics similar to *F*. *tularensis* with regards to its life cycle within macrophages and pathogenicity in mice (Rohmer et al., 2007; Kingry and Petersen, 2014). Therefore, *F*. *novicida* has been used as the model bacterium for research on *Francisella* pathogenicity.

While *Francisella* research has a long history, the detailed molecular mechanisms of infection by *Francisella* subspecies remain unknown. *Francisella* can replicate in immunocompetent cells, such as macrophages, neutrophils, and dendritic cells, which are essentially responsible for the elimination of pathogens from the body (Santic et al., 2006). To survive inside host cells, *Francisella* species employ various strategies. *Francisella* enters these cells *via* phagocytosis, escapes digestion by phagolysosomes and autophagosomes, and finally replicates in the cytoplasm (Chong et al., 2008; Miller and Celli, 2016). Further, *Francisella* suppresses or evades host pattern recognition receptors (PRRs), which usually initiate the innate immune response to exclude pathogens (Jones et al., 2011; Gillette et al., 2014; Putzova et al., 2017). Although some of the factors responsible for *Francisella*’s immunosuppressive abilities were identified in previous research (Platz et al., 2010; Nallaparaju et al., 2011; Mahawar et al., 2012), the detailed mechanisms involved in *Francisella*-mediated immunosuppression remain to be elucidated.

*Francisella* grows in nutrient-limited host cells, and this nutrient limitation is closely related to its pathogenicity (Best and Abu Kwaik, 2019). To date, a lot of genes responsible for the uptake or biosynthesis of nutrients, such as amino acids, carbon, vitamins, and bases, were reported as crucial factors for the intracellular replication of *Francisella* (Meibom and Charbit, 2010; Santic and Abu Kwaik, 2013; Feng et al., 2014). Especially, nucleotide biosynthesis is essential for the survival and virulence of bacterial pathogens, including intracellular bacteria, such as *Salmonella*, *Listeria*, *Brucella*, and *Francisella* (Goncheva et al., 2022). Since pyrimidine nucleotides are essential for all organisms, almost all bacterial species have a *de novo* pyrimidine biosynthesis pathway, which has highly conserved enzymatic steps. The *de novo* pyrimidine biosynthesis pathway for synthesizing uridine 5′-monophosphate consists of six steps and employs enzymes encoded by *carA*/*B* and *pyrB*-*F* (Turnbough Charles and Switzer Robert, 2008). The *pyrC* gene encodes a putative dihydroorotase that converts carbamoyl N-Carbamoyl-L-aspartate into 4,5-dihydroorotate in the *Francisella* pyrimidine biosynthesis pathway. Although some reports suggest pyrimidine biosynthesis is important for the intracellular growth of *Francisella*, it is not well understood how pyrimidine biosynthesis is involved in immunosuppression by *Francisella* (Qin and Mann, 2006; Horzempa et al., 2010).

In this study, we performed large-scale screening of a *F*. *novicida* transposon mutant library to search for the key factors involved in the immunosuppression mechanisms of *Francisella*. We identified *pyrC* as a novel *F. novicida* factor suppressing host innate immune responses and evaluated the immunological characteristics of host cells infected with *F*. *novicida*.

## Materials and methods

### Bacterial strains and culture conditions

*F. novicida* U112 was obtained from the Pathogenic Microorganism Genetic Resource Stock Center (Gifu University, Gifu, Japan). *F. novicida* was cultured aerobically at 37°C in a chemically defined medium (CDM) (Nagle et al., 1960) or brain heart infusion broth (Becton, Dickinson and Company, NJ, USA) supplemented with 0.1% cysteine (BHIc) (Mc Gann et al., 2010) or BHIc containing 1.5% agar (Wako Laboratory Chemicals, Osaka, Japan). All experiments were conducted in compliance with the institutional biosecurity guidelines and were approved by Yamaguchi University.

### Cell culture

Human monocytic U937 cells were grown in Roswell Park Memorial Institute (RPMI) 1640 medium (Sigma-Aldrich, St. Louis, MO, USA) supplemented with 10% heat-inactivated fetal bovine serum at 37°C in an atmosphere containing 5% CO_2_.

### Plasmid construction, transformation, and transfection

**Supplementary Table 1** lists the primer sets and templates used to construct the plasmids used in this study. Polymerase chain reaction (PCR) was performed using KOD-Plus-Neo polymerase (Toyobo, Osaka, Japan), and ligation was performed using an In-Fusion HD Cloning Kit (Takara Bio, Shiga, Japan). Plasmids were used to transform *F. novicida via* electroporation. Specifically, bacterial cells were suspended in 0.5 M sucrose with 2 μg of plasmid DNA and were electroporated using a Bio-Rad micropulser (Bio-Rad, Hercules, CA, USA) at 3.0 kV, 10 µF, and 600 Ω with a 0.2 cm cuvette. The transformants were pre-incubated in BHIc medium overnight. To select transformed bacteria, the pre-incubated bacteria were cultured on BHIc agar plates containing 30 μg/ml kanamycin or 2.5 μg/ml chloramphenicol.

### Construction of a transposon mutant library

The transposon mutant library was constructed using the Ez-Tn5 transposon system (Epicentre Lucigen, Madison, WI, USA), as previously reported (Nakamura et al., 2019). Briefly, the multiple cloning site of pMOD3 was linearized by digestion with Hind III and EcoRI, and the kanamycin resistance cassette of pKEK1140 (Rodriguez et al., 2008) was ligated into the Hind III and EcoRI sites to generate pMOD3-FtKm. The transposon moiety of pMOD3-FtKm was amplified by PCR, purified, mixed with transposase according to the manufacturer’s instructions, and then used to transform *F. novicida via* electroporation. Transformed bacteria were cultured on BHIc plates containing 30 μg/ml kanamycin.

### Sequence analysis of transposon mutants

pMOD3 harbors the *E. coli* R6Kγ origin of replication. The genomes of *F*. *novicida* transposon mutants were purified using a PureLink Genomic DNA Mini Kit (Thermo Fisher Scientific, MA, USA) and digested with a combination of restriction enzymes, including XhoI, BglII, EcoRI, SalI, NotI, and BamHI. The ends of the digested DNAs were then blunted using a DNA Blunting Kit (Takara Bio) and ligated using Ligation High Ver. 2 (Toyobo). The ligated DNA was used to transform λpir Chemically Competent *E. coli* (Thermo Fisher Scientific). The transformed *E. coli* were selected for kanamycin resistance, and plasmid DNAs were purified. Sequence analysis was performed using the primer described in the manufacturer’s instructions for the Ez-Tn5 transposon system.

### Construction of *F. novicida* mutants

The *dotU* homolog (*FTN_1316*) deletion mutant (Δ*dotU*) was previously constructed (Shimizu et al., 2019) through group II intron insertion using a TargeTron Gene Knockout System (Sigma-Aldrich), which was modified for *Francisella* species (Rodriguez et al., 2008). The *pyrC* gene (*FTN_0024*) deletion mutant (Δ*pyrC*) was generated *via* homologous recombination using the *Francisella* suicide vector pFRSU (Shimizu et al., 2019). The upstream and downstream regions of *pyrC* (1.5 kbp each) were cloned into the BamHI site of pFRSU to generate pFRSU-pyrC. The pFRSU-pyrC vector (2 μg) was used to transform *F. novicida*; transformants were selected on BHIc plates containing 30 μg/ml kanamycin. Isolated bacteria were cultured in BHIc without antibiotics overnight and then plated on BHIc plates containing 5% sucrose. The deletion of the *pyrC* gene was confirmed *via* PCR.

### Green fluorescent protein- and PyrC-expressing *F. novicida* strains

A GFP-expressing plasmid, pOM5-GFP, was constructed according to published procedures (Shimizu et al., 2019). The *F. novicida* chromosomal *pyrC* gene was cloned into pOM5 to generate pOM5-pyrC. To construct GFP-expressing strains and *pyrC* complemented strains, pOM5-pyrC and pOM5-GFP were used to transform the wild-type strain or the Δ*pyrC* mutant strain of *F. novicida via* electroporation.

### Intracellular growth assay

U937 cells (1 × 10^5^ cells/well) were incubated in a 48-well tissue culture plate with 100 nM phorbol myristate acetate (PMA) for 48 h. Then, *F. novicida* strains were added at a multiplicity of infection (MOI) of 1. Next, the plates were centrifuged for 10 min at 300 × *g* and incubated for 1 h at 37°C. The cells were then washed three times with RPMI 1640 medium, and extracellular bacteria were killed with gentamicin at 50 μg/ml for 1 h. The cells were then incubated in fresh medium at 37°C for the indicated time durations in figure legends. To measure intracellular growth, the cells were washed with PBS and then lysed with 0.1% Triton X-100 in CDM. The CFUs were determined on BHIc agar plates by plating serial dilutions of cultures.

### Fluorescence microscopy

U937 cells (1 × 10^5^ cells/well) were incubated with 100 nM PMA for 48 h on 12 mm glass coverslips in 24-well tissue culture plates. GFP-expressing *F. novicida* strains were infected at an MOI of 1. Plates were then centrifuged for 10 min at 300 × *g* and incubated for 1 h at 37°C. The cells were washed three times with RPMI 1640 medium, and extracellular bacteria were eliminated using gentamicin at 50 μg/ml for 1 h. The cells were then incubated in fresh medium at 37°C for the indicated time durations in figure legends. Cells were fixed with 4% paraformaldehyde at room temperature for 30 min. A FluoView FV1000 confocal laser scanning microscope (Olympus, Tokyo, Japan) was used to obtain images of the cells.

### RNA isolation and qPCR analysis

U937 cells (4 × 10^5^ cells/well) were incubated in a 12-well tissue culture plate with 100 nM PMA for 48 h. The medium was exchanged with fresh pre-incubated RPMI 1640 medium one-hour prior to infection. Cells were infected with *F. novicida* strains at MOI = 1 or stimulated with 100 ng/ml of lipopolysaccharide (LPS) derived from *E. coli* (O127:B8) (Sigma-Aldrich), or 10 ng/ml 2′3′-cGAMP (InvivoGen, CA, USA). The plates were centrifuged for 10 min at 300 × g and incubated for indicated time the indicated time durations in figure legends. Cells were carefully washed twice with PBS, and total RNA was collected using NucleoSpin RNA kit (Takara Bio). RNA was quantified by determining absorption at 260 nm using NanoDrop 2000 (Thermo Fisher Scientific). Next, qPCR was performed using the RNA-direct Realtime PCR Master Mix (Toyobo) with an RNA concentration of 50 ng per 20 μl reaction. The *HPRT1* amplicon was used as an endogenous control to normalize all mRNA expression data. The relative expression levels of genes in various conditions compared with those in the BHIc medium-treated control were calculated using the relative quantification method (∆∆Ct method) (Pfaffl, 2001). Used primer sets are shown in **Table S1**.

### ELISA

U937 cells (1 × 10^5^ cells/well) were pre-incubated in a 48-well tissue culture plate with 100 nM PMA for 48 h. After exchanging the medium with pre-incubated fresh RPMI medium 1 h prior to infection, cells were infected with bacterial strains (*F. novicida* transposon mutant strains or deletion mutant strains) at an MOI of 1 or stimulated with 100 ng/ml of LPS derived from *E. coli* (O127:B8) or 10 ng/ml of 2′3′-cGAMP (InvivoGen). Heat-inactivation of each strain was performed by incubating the bacterial suspension in a heat block at 90°C for 5 min. In the case of STING inhibition, a STING-specific inhibitor, H-151 was used (Haag et al., 2018). U937 cells were treated with H-151 (final concentration 0.5 μM) or the same volume of DMSO 2 h prior to infection. After incubation for the indicated time durations in figure legends, concentrations of tumor necrosis factor (TNF)-α and interleukin (IL)-1β in the supernatants were measured using ELISA MAX Standard Kit (Biolegend, CA, USA) according to the manufacturer’s instructions. Interferon (IFN)-β in the supernatants were measured using VeriKine Human Interferon Beta ELISA Kit (PBL Assay Science, Piscataway, NJ, USA).

### Statistical analysis

Student’s *t* test or multiple comparisons using the Tukey–Kramer test and Dunnett’s test were used to evaluate the significance of differences compared with the wild-type strain; *P* < 0.05 indicates a significant difference.

## Results

### Ten genes were identified as immunosuppressive factors of *F*. *novicida*

**Figure 1:**
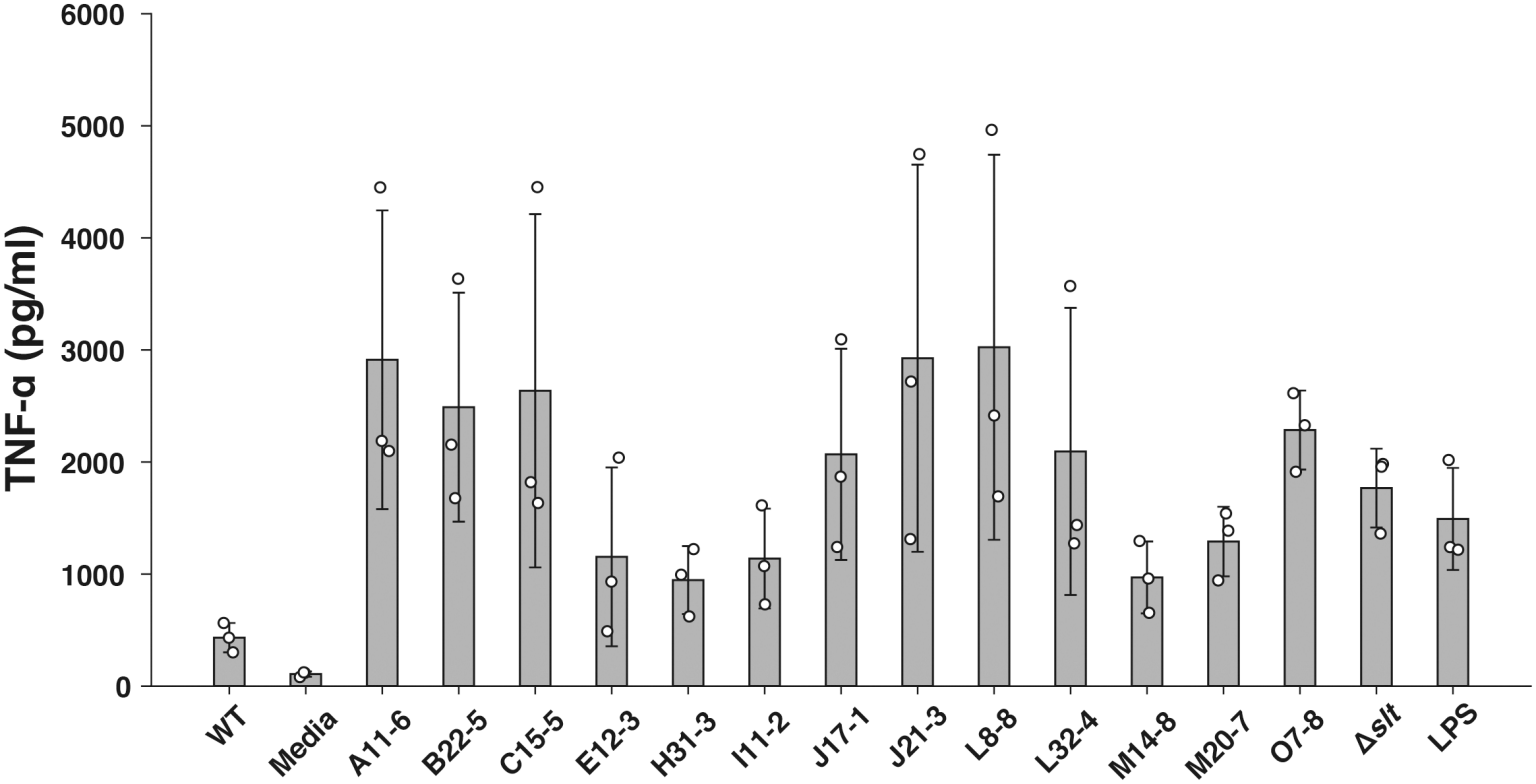
Screening of the F. novicida transposon mutant library using a TNF-α ELISA assay. U937 cells were infected with *F. novicida* transposon mutants at an MOI of 1 or stimulated with 100 ng/ml of *E*. *coli* LPS- or same volume BHIc-containing medium. After 5 h of infection, TNF-α released into the cell supernatant was measured by ELISA. Data shows averages and standard deviations from three independent experiments. Transposon mutant strains that exhibited a 1.5-fold increase in TNF-α production compared with the wild-type strain are shown.

To identify novel immunosuppressive factors of *F*. *novicida* in human macrophages, we expanded a previously constructed *F*. *novicida* transposon mutant library consist of 750 strains (Nakamura et al., 2019) up to 3552 strains. TNF-α is a cytokine produced through a broad range of innate immune signaling pathways, including Toll-like Receptor (TLR) 4- and TLR2-mediated pathways, and is reported to be suppressed by *Francisella* infection (Telepnev et al., 2003; Butchar et al., 2008). To identify genes responsible for immunosuppression by *F. novicida*, U937 cells were infected with a mutant library, and the transposon mutants inducing excessive TNF-α production compared with the wild-type strain were selected through ELISA using the culture supernatant. In the 1st screening, the cut-off was set to a 1.5-fold increase in TNF-α production. In the 2nd screening, U937 cells were infected with mutant strains selected *via* the first screening, and their TNF-α production was measured three times. Finally, 13 mutants that increased TNF-α production in U937 cells were identified (**Figure 1**). The Δ*slt* mutant strain, lacking the gene encoding soluble lytic transglycosylase, was used as a positive control (Nakamura et al., 2019). To determine the genes responsible for *Francisella*-mediated immunosuppression, the transposon insertion sites of the selected mutant strains were evaluated by sequence analysis; 10 unique genes were identified (**Table 1**). In this study, we focused on *pyrC* (*FTN_0024*), the gene putatively encoding dihydroorotase, and the effect of this gene on immunosuppression by *Francisella*.

**Table 1:** The results of screening and sequence analysis of transposon mutants.

Strain	TNF-α (pg/ml) ± S.D.	Locus	Name	Putative protein
Wild-type (WT)	432.39 ± 131.00	–	–	–
A11-6	2912.36 ± 1332.87	*FTN_0756*	*fopA*	OmpA family protein
B22-5	2489.22 ± 1021.12	*FTN_1641*	*ampG*	Major Facilitator Superfamily protein
C15-5	2635.81 ± 1576.51	*FTN_1286*	*mltA*	MltA specific insert domain protein
E12-3	1153.80 ± 797.89	*FTN_1199*	*capA/B*	Poly-gamma-glutamate system family protein
H31-3	946.36 ± 303.20	*FTN_0757*	*cas9*	CRISPR-associated endonuclease Cas9
I11-2	1138.33 ± 445.28	*FTN_1548*	*yfgL*	Outer membrane assembly lipoprotein YfgL
J17-1	2068.55 ± 942.58	*FTN_0917*	*dacB*	D-alanyl-D-alanine carboxypeptidase/D-alanyl-D-alanine-endopeptidase
J21-3	2925.88 ± 1726.95	*FTN_0496*	*slt*	Transglycosylase SLT domain protein
L8-8	3024.12 ± 1718.24	*FTN_0496*	*slt*	Transglycosylase SLT domain protein
L32-4	2094.21 ± 1281.19	*FTN_0611*	*kdsA*	3-deoxy-D-manno-octulosonic acid 8-phosphate synthase
M14-8	970.04 ± 320.70	*FTN_0757*	*cas9*	CRISPR-associated endonuclease Cas9
M20-7	1290.48 ± 310.36	*FTN_0024*	*pyrC*	Dihydroorotase, multifunctional complex type domain protein
O7-8	2285.06 ± 352.58	*FTN_0496*	*slt*	Transglycosylase SLT domain protein
Δ*slt*	1767.43 ± 351.55	*FTN_0496*	*slt*	Transglycosylase SLT domain protein
LPS (O127:B8)	1491.96 ± 455.53	–	–	–

### The *pyrC* deletion mutant evokes the innate immune responses of host U937 macrophage cells

**Figure 2:**
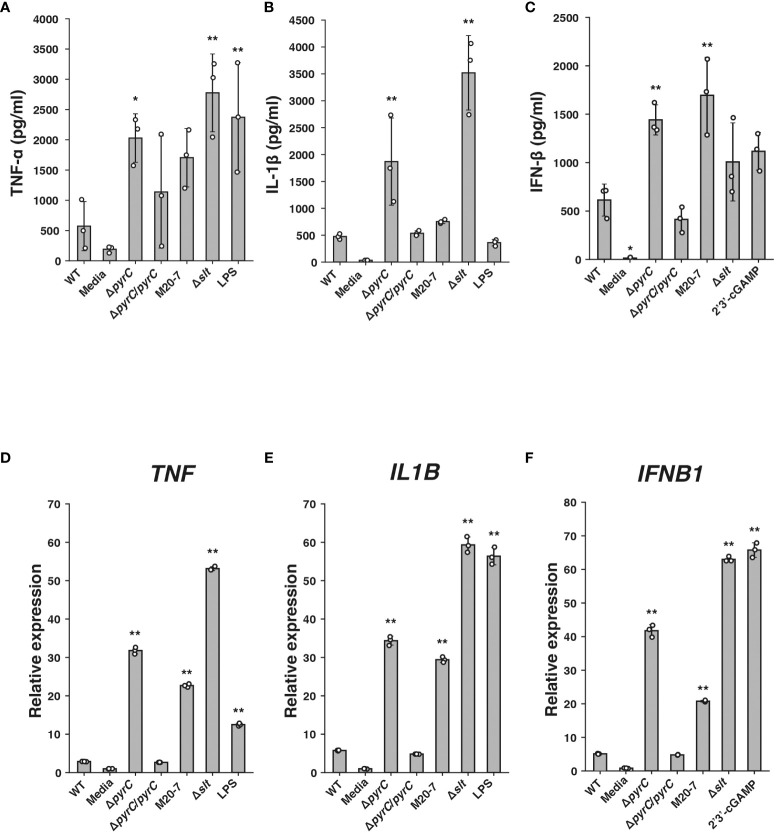
Cytokine induction by the *F novicida pyrC* deletion mutant. U937 cells were stimulated with 100 ng/ml of *E coli* LPS-containing medium or 10 ng/ml 2′3′-cGAMP- or same volume BHIc-containing medium or infected with *F novicida* strains (MOI = 1). Cell supernatants were collected and concentrations of TNF-α at 5 h post infection (p.i.) **(A)**, IL-1β at 12 h p.i. **(B)**, and IFN-β at 24 h p.i. **(C)** were measured by ELISA. Total RNA was collected, and mRNA expression of *TNF* at 5 h p.i. **(D)**, *IL1B* at 5 h p.i. **(E)**, and *IFNB1* at 12 h p.i. **(F)** was measured by qPCR; relative expression normalized to that in the same volume BHIc-containing medium treatment control are shown. Data shows averages and standard deviations from three independent experiments. Differences compared with the wild-type strain were determined by Dunnett’s multiple comparison and are indicated by asterisks **P < 0.01, *P < 0.05.

To estimate the effect of *pyrC* in immunosuppression by *F*. *novicida*, we constructed a Δ*pyrC* mutant strain of *F*. *novicida via* homologous recombination. ELISA showed that the Δ*pyrC* mutant strain induced significantly higher levels of TNF-α production in the cell culture supernatant of U937 cells than the wild-type strain (**Figure 2A**). Although not significantly different, the *pyrC* complemented strain tended to show decreased levels of TNF-α induction compared with the Δ*pyrC* mutant strain. The *pyrC* complemented strain showed equivalent levels of TNF-α induction to the wild-type strain (**Figure 2A**). Next, we measured the induction of IL-1β and IFN-β, which are important cytokines for *Francisella* infection, in the Δ*pyrC* mutant strain (Gavrilin and Wewers, 2011). As in the case of TNF-α, the Δ*pyrC* mutant strain-infected U937 cells showed significantly higher levels of IL-1β and IFN-β production than the wild-type strain, and *pyrC* complementation decreased these levels to that observed upon infection with the wild-type strain (**Figures 2B, C**). The mRNA expression levels of *TNF*, *IL1B*, and *IFNB1* in *Francisella*-infected U937 cells were also examined using real-time PCR. Similar results to those obtained *via* ELISA were observed for the mRNAs of all these genes (**Figures 2D–F**). These results indicate that the Δ*pyrC* mutant strain strongly evokes host innate immune responses compared to the wild-type strain, suggesting that *pyrC* is a critical factor for the immunosuppression of *F*. *novicida*.

### The *pyrC* gene is important for the intracellular growth of *F*. *novicida* in U937 cells

**Figure 3:**
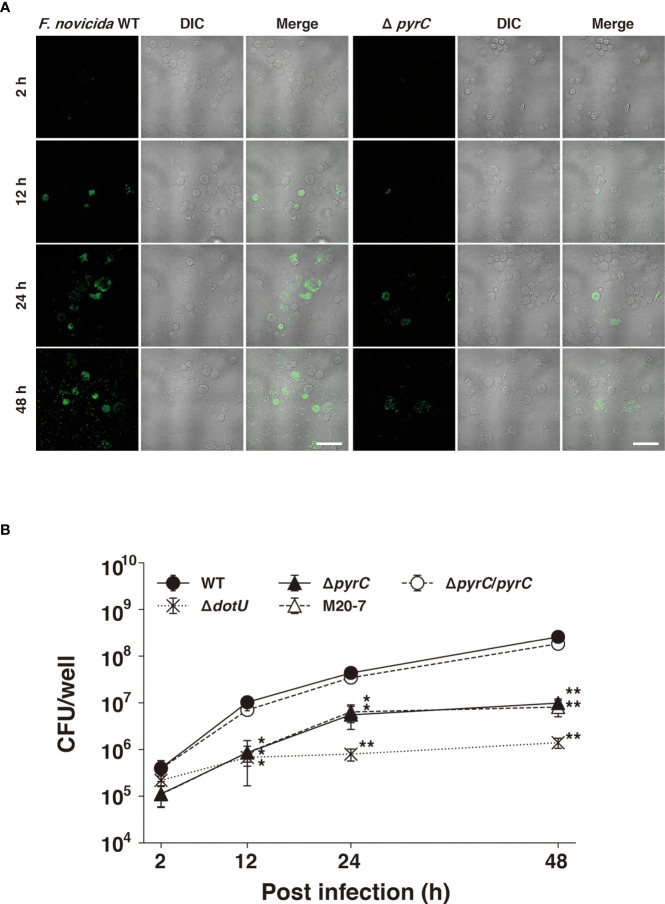
Intracellular growth of the *pyrC* deletion mutant. **(A)** U937 cells were infected with GFP-expressing *F novicida* strains at an MOI of 1 and treated with 50 μg/ml of gentamicin for 1 (h) At 2–48 h post infection, the cells were fixed and observed by FV1000 confocal laser scanning microscopy. Scale bar: 20 μm. **(B)** U937 cells were infected with *F novicida* strains at an MOI of 1 and treated with 50 μg/ml of gentamicin for 1 (h) At indicated time points post infection, cells were disrupted with 0.1% Triton X-100 and plated on BHIc agar; colony forming units were then counted. Data shows averages and standard deviations from three independent experiments. Differences compared with the wild-type strain were determined by Dunnett’s multiple comparison and are indicated by asterisks **P < 0.01, *P < 0.05.

Next, we examined whether Δ*pyrC* is involved in the intracellular growth of *F. novicida*. The Δ*pyrC* mutant strain entered the stationary phase slightly earlier than wild-type strain in BHIc medium and failed to grow in CDM (**Supplementary Figure1**). The GFP-expressing wild-type strain of *F. novicida* grew intracellularly in U937 cells from 2 to 48 h post infection, while the GFP-expressing Δ*pyrC* mutant strain did not show remarkable intracellular growth during the same period through fluorescence microscopy (**Figure 3A**). To support this finding, the number of Δ*pyrC* and transposon mutant strain cells were significantly decreased to approximately 1/10th that of wild-type strain cells within U937 cells. On the contrary, the Δ*pyrC* mutant strain showed higher intracellular growth compared to the strain deficient in the type VI secretion system (Δ*dotU*), which was used as a negative control. The complemented strain restored the ability of the Δ*pyrC* mutant strain to grow intracellularly (**Figure 3B**). These results suggest that *pyrC* is not essential but is important for the intracellular growth of *F*. *novicida* in host cells.

### TNF-α induction by the Δ*pyrC* mutant strain is abolished by heat treatment

**Figure 4:**
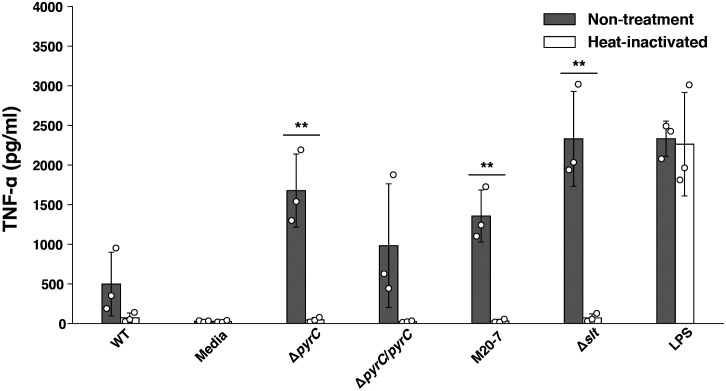
TNF-α induction by heat-inactivated *F. novicida pyrC* deletion mutant. U937 cells were stimulated with 100 ng/ml of *E*. *coli* LPS- or same volume BHIc-containing medium or infected with *F. novicida* strains (MOI = 1). Heat inactivation of the bacterial suspension was performed at 90℃ for 5 min. At 5 h post infection, cell supernatants were collected, and TNF-α concentrations were measured by ELISA. Data shows averages and standard deviations from three independent experiments. The differences among various conditions were analyzed by Tukey–Kramer multiple comparison and are indicated by asterisks **P < 0.01.

Host cells infected with bacteria produce TNF-α due to the recognition of various bacterial ligands, such as LPS, peptidoglycan, and nucleotides, by PRRs (Kawai and Akira, 2006; Li and Wu, 2021). To identify Δ*pyrC* mutant strain ligands responsible for the induction of TNF-α in the Δ*pyrC* mutant strain infected U937 cells, U937 cells were treated with heat-inactivated *F. novicida* mutant strains. TNF-α induction by all heat-inactivated *F*. *novicida* strains, including the Δ*pyrC* mutant strain, was decreased to the same level as that by the negative control; however, LPS retained the ability to induce TNF-α with or without heat inactivation (**Figure 4**). This result suggests that TNF-α production by *F. novicida* strains, including the Δ*pyrC* mutant strain, is induced through biological activities of *F. novicida*, such as internalization by or proliferation in host cells, and not through heat-stable ligands, such as LPS, peptidoglycan, and nucleotides.

### IFN-β induction in U937 cells infected with the Δ*pyrC* mutant strain is mediated by the STING pathway

**Figure 5:**
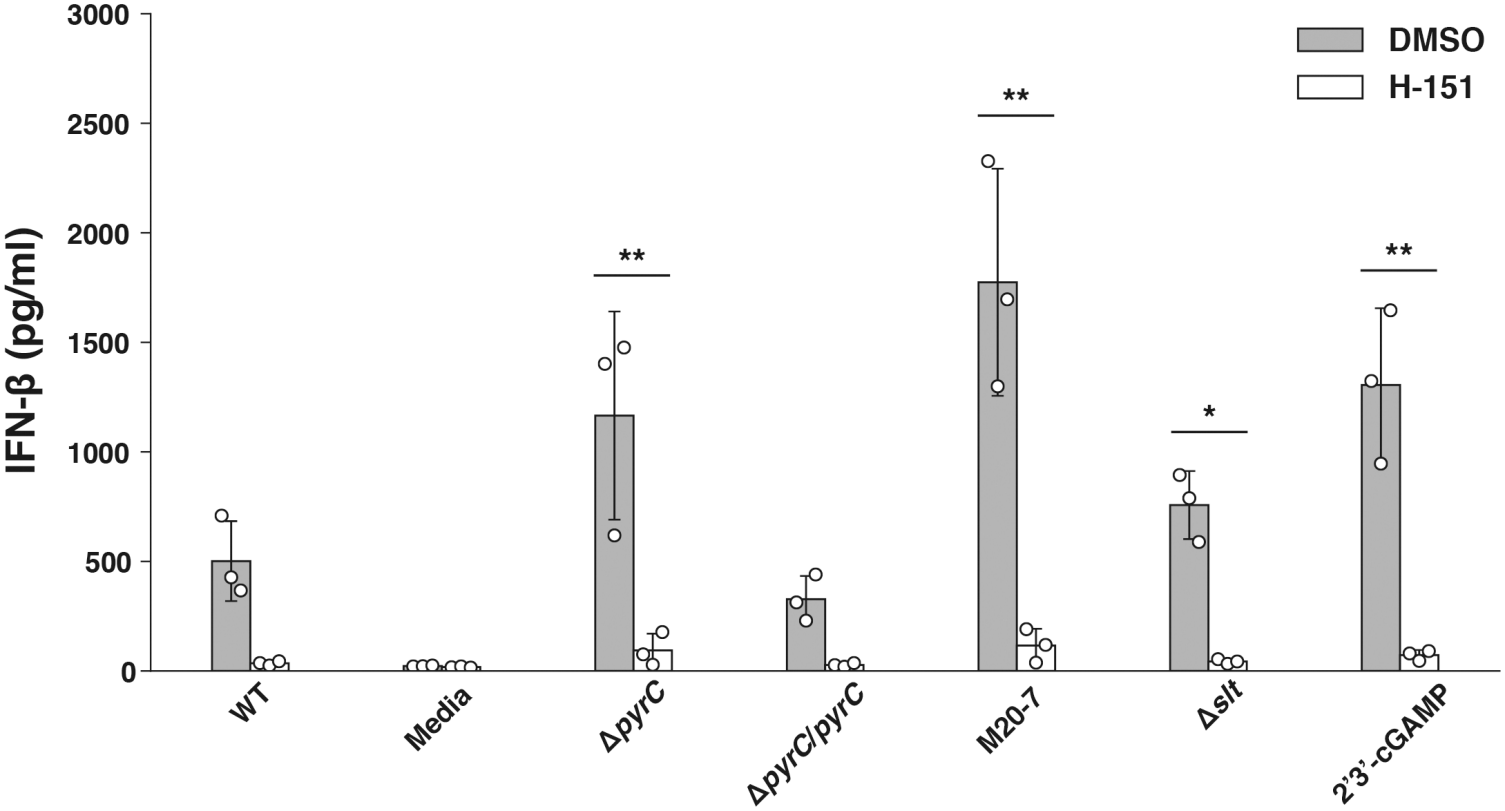
IFN-β induction by the *F. novicida pyrC* deletion mutant under STING inhibition. U937 cells were treated the H-151 STING inhibitor 2 h prior to infection and then stimulated with 10 ng/ml 2′3′-cGAMP, BHIc medium or infected with *F. novicida* strains (MOI = 1). At 24 h post infection, cell supernatants were collected, and concentrations of IFN-β were measured by ELISA. Data shows averages and standard deviations from three independent experiments. The differences among various conditions were determined by Tukey–Kramer multiple comparison and are indicated by asterisks **P < 0.01, *P < 0.05.

Because IFN-β production is induced by recognition of cytosolic DNA by the cyclic-di-nucleotide sensor STING (Jones et al., 2010; Storek et al., 2015) pathway, we next examined whether *F. novicida pyrC* affects the STING pathway. U937 cells treated with a STING inhibitor H-151 were infected with the Δ*pyrC* mutant strain, and the IFN-β levels induced in them were measured using ELISA. H-151 showed no significant effect on the growth of both the wild-type and the Δ*pyrC* mutant strain in BHIc medium (**Supplementary Figure 2**). IFN-β levels in the supernatant of H-151-treated U937 cells infected with the Δ*pyrC* mutant or transposon mutants and stimulated with the STING agonist 2′3′-cGAMP were significantly decreased compared to those in the supernatant of the DMSO control cells (**Figure 5**). Contrarily, H-151-treated U937 cells showed no significant difference in IFN-β induction upon infection with Δ*pyrC* mutant or transposon mutants compared to the wild-type and *pyrC* complemented strains. These results indicate that *pyrC* is involved in the suppression of IFN-β through the STING pathway.

## Discussion

Intracellular bacteria, including *Francisella*, have refined their strategy to escape the host immune system and survive in host cells. To date, the importance of immunosuppression and immune evasion in *Francisella* infection has been well recognized, but their detailed mechanisms are poorly understood. To our knowledge, a large-scale gene screening of *Francisella* mutants focusing on *Francisella* immunosuppressive properties has not been performed yet. Therefore, here, we developed a transposon mutant library of *F*. *novicida* consisting of 3552 mutants. This library seems to cover the 1731 protein coding genes of the *F*. *novicida* U112 strain (Rohmer et al., 2007). Among the 3552 mutant strains in our library, strains that induced higher levels of host immune response than the wild-type *F*. *novicida* strain were isolated. Ten genes were determined to be immunosuppression-related. Among these 10 genes, four genes, *FTN_0756* (*fopA*), *FTN_1286* (*mltA*), *FTN_0757* (*cas9*), and *FTN_0496* (*slt*), were previously reported as immunosuppressive factors of *Francisella* (Peng et al., 2011; Sampson et al., 2013; Nakamura et al., 2019; Nakamura et al., 2021), ensuring the reliability of the screening method employed in this study. The remaining 6 genes, *FTN_1641* (*ampG*), *FTN_1199* (*capA*/*B*) *FTN_1548* (*yfgL*), *FTN_0917* (*dacB*), *FTN_0611* (*kdsA*), and *FTN_0024* (*pyrC*), were newly identified as *Francisella* immunosuppressive factors in this study. Because *ampG*, *capA*/*B*, *yfgL*, *dacB*, *and kdsA* are involved in creating the structure of bacterial cells, disruption of these genes can result in the leakage of ligands for intracellular receptor inflammasomes, such as LPS, peptidoglycan, and nucleotides (Peng et al., 2011; Man and Kanneganti, 2015; Tsuchiya, 2020). In contrast, the gene *pyrC* putatively encodes dihydroorotase, which converts carbamoyl N-Carbamoyl-L-aspartate into 4,5-dihydroorotate through the *Francisella* pyrimidine biosynthesis pathway (Turnbough Charles and Switzer Robert, 2008). It was previously reported that another pyrimidine biosynthesis gene *pyrF* is involved in immune suppression by the *F*. *tularensis* LVS (Horzempa et al., 2010). Although these results indicate that pyrimidine biosynthesis pathway is strongly related to immunosuppression by *Francisella*, the detailed mechanisms through which the pyrimidine biosynthesis pathway affects immunosuppression by *Francisella* remain unclear. Therefore, we focused on *pyrC* in this study. In this screening method, *pyrF* was not identified as immune suppressive factor. This may indicate that 3552 mutants are not enough to cover the 1731 protein coding genes of *F*. *novicida* or there is a bias in the insertion site of transposon.

To confirm the effects of *pyrC* on immunosuppression, a *pyrC* deletion mutant was generated. The Δ*pyrC* mutant strain induced a high level of TNF-α production in infected U937 macrophage cells compared to that in the wild-type strain, and the complemented strain restored the immunosuppressive property of *F. novicida*. TNF-α is produced through a broad range of innate immune signaling pathways, including TLR signaling pathways activated by various bacterial ligands, such as LPS, peptidoglycan, and nucleotides (Kawai and Akira, 2006; Li and Wu, 2021). In host cells infected by *Francisella*, the production of inflammatory cytokines, such as TNF-α and IL-6, is induced by the recognition of *Francisella* by TLR2, followed by the recognition of *Francisella* DNA by TLR 9 (Jones et al., 2012). Because *pyrC* is related to pyrimidine biosynthesis, it may suppress immune responses by modifying nucleotides recognized by TLRs, such as TLR9. However, as discussed below, heat-inactivated *F. novicida* strains, including the Δ*pyrC* mutant strain, failed to induce TNF-α production, indicating that biological activities of *F. novicida*, such as its phagosomal escape, are necessary for it to induce TNF-α and other cytokines.

Our data also revealed that the disruption of the pyrimidine biosynthesis pathway by *F*. *novicida* induces higher levels of IL-1β and IFN-β production in U937 macrophage cells. IL-1β is secreted when its precursor is expressed through the activation of TLR or type I IFN signaling followed by cleavage with caspase-1 activated through recognition by inflammasomes (Henry et al., 2007; Man and Kanneganti, 2015). In *F. novicida* infections, infected host cells exhibit robust inflammasome activation and IL-1β secretion compared with *F*. *holarctica* LVS and *F*. *tularensis* Schu S4 infections (Jones et al., 2011; Ghonime et al., 2015). In this study, we found that IL-1β production was suppressed in host cells infected by wild-type *F. novicida* and increased in host cells infected with the Δ*pyrC* mutant strain. This result is inconsistent with those of previous reports by Horzempa’s and Schulert, which indicate that several genes of *F*. *tularensis* Schu S4 and *F*. *holartica* LVS strains are related to the suppression of IL-1β production (Schulert et al., 2009; Horzempa et al., 2010). Our results, however, show that the expression of IL-1β mRNA was suppressed upon host cell infection by the wild-type strain of *F. novicida* and increased upon host cell infection with the Δ*pyrC* mutant strain. Taken together with the fact that the IL-1β precursor is expressed through TLR or type I IFN signaling, the suppression of IL-1β secretion by *pyrC* cannot be attributed to the inhibition of inflammasomes but to the inhibition of TLR or type I IFN signaling.

Type I IFNs are secreted through STING-related pathways. The cGAS-STING pathway, known as an intracellular DNA sensor, recognizes bacterial DNA and induces type I IFNs (Ishikawa and Barber, 2008). STING is also activated by direct recognition of cyclic-di-nucleotides released by bacteria (Barber, 2015). Type I IFN secretion resulting from *Francisella* infection exacerbates the infection. It was reported that type I IFNs suppress host immunity by inhibiting IL-17A expression of γδT cell, and deficiency in type I IFN-related molecules, such as cGAS, STING, IFNAR1, and IRF3, in mice result in resistance to *Francisella* infection (Henry et al., 2007; Henry et al., 2010; Storek et al., 2015). However, bacterial factors involved in the modulation of host type I IFNs and their mechanisms of action are incompletely understood. In this study as well, INF-β was suppressed in U937 cells infected with *F. novicida*, and *pyrC* was related to this suppression. To elucidate the mechanisms involved in IFN-β suppression, the STING inhibitor H-151 was used. H-151 decreased the induction of IFN-β in U937 cells infected by the Δ*pyrC* mutant strain, indicating that *pyrC* is involved in the suppression of pathways related to STING. IFN-β production through STING in *Francisella* infected cells has been observed in human cell line and mouse infection models (Jones et al., 2010; Nandakumar et al., 2019). Several studies revealed that IFN-β secretion by the STING-dependent detection of *F*. *novicida* in host cell cytoplasm boosts inflammasome activation, IL-1β release, and pyroptosis by promoting gene expression of gamma guanylate proteins, ZBP1, and pyrin (Henry et al., 2007; Man et al., 2015; Meunier et al., 2015; Lee et al., 2021). In our investigation, IFN-β and IL-1β were induced in U937 cells infected with the Δ*pyrC* mutant strain, not only at the protein level, but also at the mRNA level. Because STING pathway activation was found to induce IL-1β mRNA expression in a type I IFN-dependent manner (Swanson et al., 2017), our findings suggest that *pyrC* is crucial for IFN-β suppression followed by the suppression of IL-1β secretion.

To determine the ligands that induce TNF-α in U937 cells infected with the Δ*pyrC* mutant strain, we treated U937 cells with heat-inactivated *Francisella* mutant strains, including the Δ*pyrC* mutant strain. TNF-α production decreased to control levels in all the heat-inactivated strains. This result indicates that TNF-α production is not caused by the recognition of *F. novicida* by heat-resistant ligands, such as LPS, peptidoglycan, and nucleotides, present outside the cells, but by biological responses of *F. novicida*, such as intracellular proliferation. A previous report on *Pseudomonas aeruginosa* indicated that uracil controls biofilm formation *via* quorum-sensing, and *P. aeruginosa* mutants lacking genes involved in uracil biosynthesis could not form biofilms (Ueda et al., 2009). A biofilm is a structured community of microbial cells in a matrix formed by extracellular polymeric substances (EPS). The EPS consist of polysaccharides, nucleic acids (extracellular DNA and RNA), proteins, lipids, and other biomolecules (Karygianni et al., 2020). It has also been shown that biofilms formed by *Mycobacterium avium* and *P*. *aeruginosa* and the extracellular DNA of these bacteria have the potential to induce TNF-α production in host cells infected by them (Rose and Bermudez, 2014; Ramirez et al., 2019). In the Δ*pyrC* mutant strain-infected U937 cells, the production of the TNF-α, IL-1β, and IFN-β cytokines was increased in response to the recognition of bacterial nucleic acids compared with that in wild-type strain-infected U937 cells. These results suggest that *pyrC* may be involved in the coordination or modification of ligands, such as extracellular DNA in host cytosol, protecting *Francisella* from TLR or STING recognition and allowing it to grow intracellularly by suppressing immune responses.

Intracellular growth is one of the most important abilities determining *Francisella*’s pathogenicity, a lot of genes involved in the intracellular growth of *Francisella* have been identified in previous studies (Qin and Mann, 2006; Su et al., 2007). PyrC is required for *de novo* pyrimidine biosynthesis (Choi and Zalkin, 1990). Mutants of *Francisella* genes involved in the pyrimidine pathway (e.g., *carA*, *pyrB*, *pyrD*, and *pyrF*) become uracil auxotrophs and show deficient growth on complete medium (Maier et al., 2006; Qin and Mann, 2006; Horzempa et al., 2010). Several reports indicate that these pyrimidine pathway-related mutants can grow within epithelial cells but not macrophage cells (Qin and Mann, 2006; Horzempa et al., 2010). In addition, Schulert et al. showed that a pyrimidine biosynthesis pathway transposon mutant of *F*. *novicida* was eliminated by monocyte-derived macrophages, in part *via* phagosomes (Schulert et al., 2009). In our study, the Δ*pyrC* mutant showed decreased growth in culture medium, and decreased but constant intracellular growth in U937 cells compared with that of the wild-type strain. *Francisella* mutants that is deficient in the phagosomal escape-related factor such as *mglA* or type VI secretion system, have no ability to induce IL-1β secretion (Mariathasan et al., 2005; Gavrilin et al., 2006; Jones et al., 2010). These results indicate that the Δ*pyrC* mutant strain can enter the host cytoplasm and grow intracellularly. Although the intracellular bacterial number of the Δ*pyrC* mutant strain was relatively low, it increased the production of cytokines compared with that in cells infected with the wild-type strain, suggesting strongly that *pyrC* contributes to the suppression of host immune responses.

In summary, we performed here, a large-scale screening to search for factors responsible for immunosuppression by *F. novicida* in human macrophage cells. Ten genes were determined to be responsible for the immunosuppression. Among them, *pyrC* was identified as a novel *F*. *novicida* immunosuppressive factor and was immunologically characterized. Although further studies are needed to elucidate the detailed mechanisms by which *pyrC* is involved in host immunosuppression by *Francisella*, research on pyrimidine metabolic pathways involving *pyrC* may provide new insight into *Francisella* immunosuppression and pathogenicity and into the mechanisms by which host cells recognize intracellular bacteria.

## Data availability statement

The raw data supporting the conclusions of this article will be made available by the authors, without undue reservation.

## Author contributions

TN contributed to laboratory analysis, investigation, and writing of the original draft. TS contributed to study conceptualization, laboratory analysis, investigation, and writing of the original draft. RI contributed to laboratory analysis. AU contributed to methodology and resources. KW contributed to laboratory analysis, investigation, and validation. MW was involved in study conceptualization, laboratory analysis, supervision, manuscript review, and manuscript editing. All authors contributed to the article and approved the submitted version.

## Funding

This work was supported by JSPS KAKENHI Grant Number 19K07556 and 22K07054 and Grant-in-Aid for JSPS Fellows Grant Number 22J20065.

## Conflict of interest

The authors declare that the research was conducted in the absence of any commercial or financial relationships that could be construed as a potential conflict of interest

## Publisher’s note

All claims expressed in this article are solely those of the authors and do not necessarily represent those of their affiliated organizations, or those of the publisher, the editors and the reviewers. Any product that may be evaluated in this article, or claim that may be made by its manufacturer, is not guaranteed or endorsed by the publisher.
