# Supplementary material for: Identification of pyrC gene as an immunosuppressive factor in Francisella novicida infection
Source: Front Cell Infect Microbiol. 2022 Oct 26;12:1027424. doi: 10.3389/fcimb.2022.1027424 (PMC9643745; doi:10.3389/fcimb.2022.1027424)
Supplement: Supplementary file 1 [file DataSheet_1.docx]

| Vector | Primer | Sequence | Template (Reference) |
| --- | --- | --- | --- |
| pMOD3-FtKm | pMOD3-kanR.FOR | CATCGTGGCCGGATCGATCTTTTGGGTTGTCACTCATCGTATT | pKEK1440 |
|  | pMOD3-kanR.REV | ATTAACCAATTCTGATTAGAAAAACTCATCGAGCATCAAATGAAACT |  |
|  | pMOD3-kanR-vector.FOR | TCAGAATTGGTTAATTGGTTGTAACACTGG | pMOD3 |
|  | pMOD3-kanR-vector.REV | GATCCGGCCACGATGCG |  |
|  |  |  |  |
| pFRSU-pyrC | FTN_0024_up1.5.FOR | GCAGCCCGGGGGATCGATTTCAACCAATACCAATAATGACCTTGTCAG | *F.novicida* genome |
|  | FTN_0024_up1.5.REV | TGATAGCATTTTTCCTCCTTTATAATCTACCCAAATTATAATGAACCG |  |
|  | FTN_0024_down1.5.FOR | GGAAAAATGCTATCAGCTACTAATGTTGTAGGGAAA |  |
|  | FTN_0024_down1.5.REV | TAGAACTAGTGGATCGGGGAATCAGCACATTAGCGATTAG |  |
|  | FTN_0024_check.FOR | ACCCACATCATCATTTTGCCA |  |
|  | FTN_0024_check.REV | GGATGAACTCCTAGCACACCA |  |
|  |  |  |  |
| pOM5-pyrC | pOM5_PyrC.FOR | AGCAAGGAGAAGTCAATGTCAAATCAAAGTTTACTTATCAAAAATGCTACGGTTGTAAATGAG | pOM5-IglC (Nakamura et al., 2019) |
|  | pOM5_PyrC.REV | TGATCTTTTCTACGGTCAAAACTCATGATTAAACTCTAAACATTGTCCTCTTTGATCGC |  |

| GENE | Primer | Sequence |
| --- | --- | --- |
| *HPRT1* | Human HRPT1.FOR | GACCAGTCAACAGGGGACAT |
|  | Human HRPT1.REV | AACACTTCGTGGGGTCCTTTTC |
|  |  |  |
| *TNF* | Human TNF.FOR | CCTCTCTCTAATCAGCCCTCTG |
|  | Human TNF.REV | GAGGACCTGGGAGTAGATGAG |
|  |  |  |
| *IL1B* | Human IL1B.FOR | AGCTACGAATCTCCGACCAC |
|  | Human IL1B.REV | CGTTATCCCATGTGTCGAAGAA |
|  |  |  |
| *IFNB1* | Human IFNB1.FOR | TTGACATCCCTGAGGAGATTAAGC |
|  | Human IFNB1.REV | TTAGCCAGGAGGTTCTCAACAATAG |

**Supplementally Table 1 Primer sets and templates.**


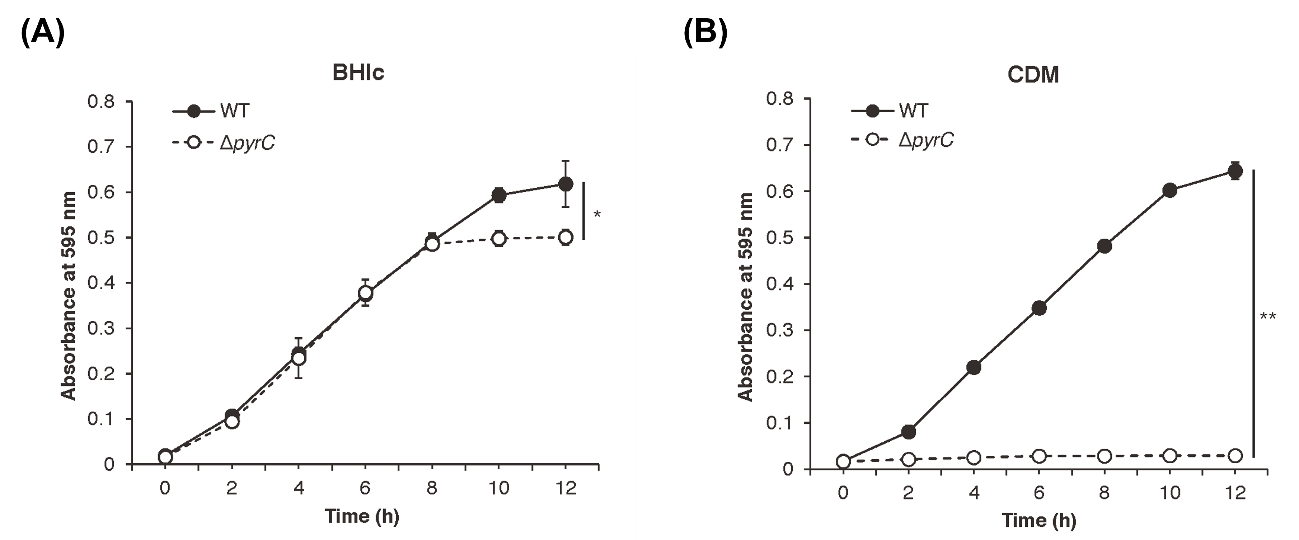


**Supplementary Figure 1. Growth of the Δ*pyrC* mutant strain in liquid medium.**

The wild-type (WT) and the Δ*pyrC* mutant strain of *F*. *novicida* were cultured in BHIc (A) or CDM medium (B). Absorbances were measured at 595 nm. Data shows averages and standard deviations from three independent experiments. Differences compared with the wild-type strain at 12 h were determined by the Student’s *t* test and significances were indicated by asterisks; ***P* < 0.01, **P* < 0.05.


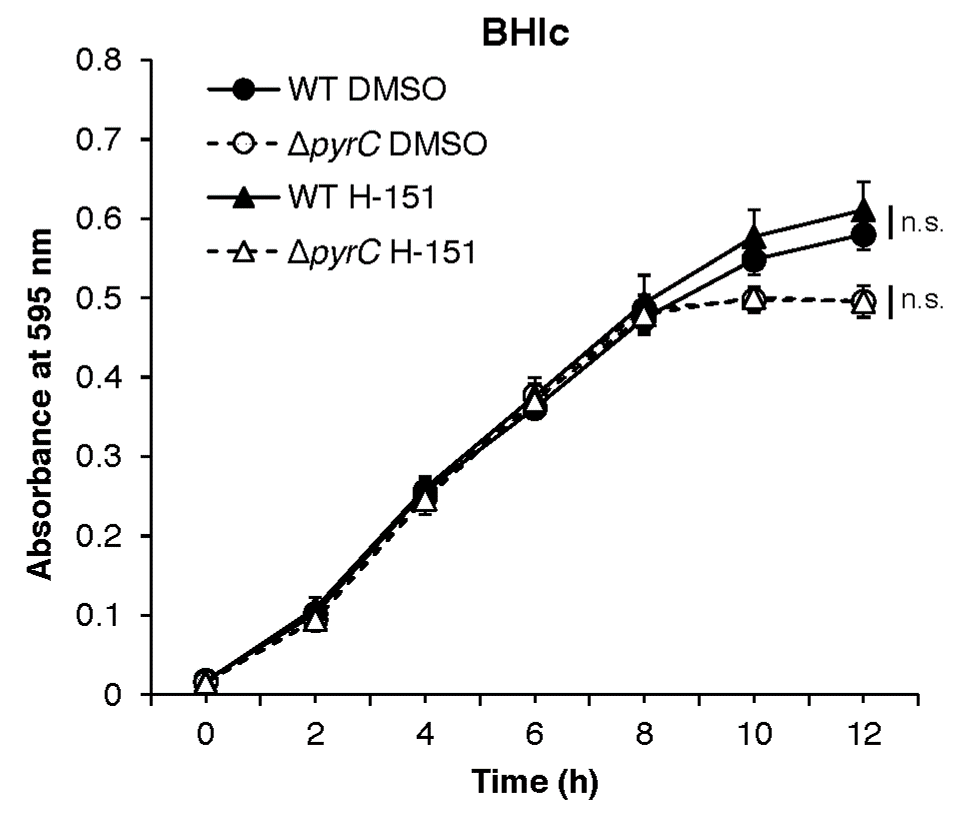


**Supplementary Figure 2. Growth of the Δ*pyrC* mutant strain in liquid medium containing H-151.**

The wild-type (WT) and the Δ*pyrC* mutant strain of *F*. *novicida* were cultured in BHIc medium containing 0.5 μM H-151 or the same volume of dimethyl sulfoxide (DMSO). Absorbances were measured at 595 nm. Data shows averages and standard deviations from three independent experiments. Each difference between DMSO and H-151 condition at 12 h was determined by the Student’s *t* test and significances were indicated by asterisks; ***P* < 0.01, **P* < 0.05, n.s. = not significant.
